# Inhibition of PDE-4 and PDE-5 Differentially Modulates Isolated Porcine Urethral Contractility

**DOI:** 10.1007/s00192-025-06102-4

**Published:** 2025-03-13

**Authors:** Eriq Burovski, Donna Sellers, Russ Chess-Williams, Iris Lim

**Affiliations:** Centre for Urology Research, Faculty of Health Sciences & Medicine, Bond University, 14 University Drive, Robina, Gold Coast, QLD 4226 Australia

**Keywords:** cAMP pathway, NO/cGMP pathway, PDE inhibitors, Stress urinary incontinence, Urethral contractility, Urethral smooth muscle

## Abstract

**Purpose/Objective:**

This study explores the role of phosphodiesterase (PDE) inhibitors (specifically PDE-4, PDE-5 and PDE-1) in modulating the contractility of the porcine urethral smooth muscle and mucosal layers.

**Methods:**

Using an organ bath setup, the effects of PDE inhibitors rolipram, roflumilast, sildenafil, tadalafil and vinpocetine (0.1 nM to 10 μm) on isolated porcine urethral mucosa-intact smooth muscle, mucosa-denuded smooth muscle and mucosal layers were investigated.

**Results:**

Our results demonstrate that PDE-4 inhibitors (rolipram and roflumilast) significantly relaxed mucosa-intact urethral smooth muscle and reduced spontaneous contraction rates in the mucosal strips. Conversely, PDE-5 inhibitors (sildenafil and tadalafil) relaxed smooth muscle tissues denuded of mucosa but required exogenous source of nitric oxide (sodium nitroprusside) for effectiveness in relaxing the mucosa-intact tissues. PDE-1 inhibitor vinpocetine exhibited negligible effects.

**Conclusion:**

The results from the study suggest a potential role of the cAMP pathway in modulating spontaneous contractions within the urethral mucosa, while the NO/cGMP pathway appears to be important in modulating urethral smooth muscle tonic contractions. These findings suggest differential roles of PDE isoenzymes in urethral tissues.

## Introduction

Stress urinary incontinence (SUI) is the most prevalent incontinence issue in adult women, with more than one in three affected, and the second most prevalent issue in adult men, causing physical, emotional, and social distress [1–4]. Underlying risk factors associated with SUI include being female, obese, a smoker, > 45 years of age and having a history of vaginal delivery [5]. With a large number of individuals being affected by SUI, the systematic expenditure on management and treatment is substantial. This is reflected in recent findings estimating the economic burden associated with SUI to be over $12 billion annually and rising within the United States [6], with costs inclusive of direct (diagnosis, treatment and routine care) and indirect costs (lost workdays and decreased productivity) [7]. Therefore, understanding the risk factors, preventative measures and treatment of SUI is vital to reduce its impact on healthcare systems and improving patients’ overall quality of life.

Current treatment options for SUI include nonconservative and conservative treatment. Nonconservative treatment options include surgical interventions such as mid-urethral slings, which provide support under the urethra when there is a rise in intra-abdominal pressure to allow for the area to remain dry [8, 9]. Conservative treatment options include off-label pharmaceutical treatment options such as serotonin-norepinephrine reuptake inhibitors and β-adrenoreceptor agonists. However, the limitations and side effects of these treatments necessitate the exploration of new pharmacological approaches and targets. Little is known about urethral smooth muscle function, and understanding it more deeply can reveal potential target pathways and mechanisms, leading to new and effective pharmacological treatments.

Phosphodiesterase (PDE) inhibitors are currently used in the management and treatment of chronic obstructive pulmonary disease, erectile dysfunction, pulmonary arterial hypertension and benign prostatic hyperplasia [10, 11]. The mechanism of action of PDE inhibitors is to enhance cyclic adenosine monophosphate (cAMP) and cyclic guanosine monophosphate (cGMP) levels by inhibiting the action of PDE isoenzymes, which are responsible for the breakdown of cAMP and cGMP [12, 13]. Studies on human and animal tissues have shown the potential for PDE inhibitors to effectively relax the smooth musculature of the bladder to cause desired results [12].

Experimental studies have reported that PDE-5 inhibitors, such as sildenafil and tadalafil have the potential to attenuate the tension of isolated porcine, rat, rabbit and human urethral smooth muscle [14–18]. These findings suggest that PDE-5 isoenzymes of the nitric oxide (NO)/cGMP pathway may have a therapeutic potential as a novel treatment target for SUI. Additionally, a previous study that has demonstrated the role of adenylyl cyclase activation and subsequent cAMP production in mouse isolated urethral rings highlights the complexity of the signalling pathways involved in urethral function [19]. A study investigating the isolated human male urethral smooth muscle tissue suggested that PDE-4 inhibitor rolipram was equal to or more efficient in relaxing contracted urethral tissues than other PDE-5 inhibitors investigated (vardenafil and sildenafil) [15]. These findings indicate that cAMP may also have a role alongside the NO/cGMP pathway in regulation of urethral contractility.

The urethra consists of multiple layers, including an epithelial mucosal layer, a lamina propria, and smooth muscle layers. The mucosal layer serves protective and sensory functions, while the smooth muscle layers play a crucial role in regulating urethral tone. Despite these structural complexities, the potential effects of inhibiting PDE isoforms within the different tissue layers of the urethra have not been investigated [20]. This gap in the current understanding underscores the need for further investigation into the distinct roles of PDE-4 and PDE-5 isoenzymes in the urethral tissue. SUI is caused by insufficient urethral closure pressure, often exacerbated by increases in intra-abdominal pressure during activities such as coughing or lifting. While PDE inhibitors are known for their smooth muscle relaxant properties, their role in SUI treatment is less clear. Relaxation of urethral smooth muscle may appear counterproductive; however, our study investigates how PDE isoenzymes modulate urethral contractility, aiming to identify specific pathways that could be therapeutically targeted. This approach could uncover nuanced mechanisms, particularly in the urethral mucosa, which may influence urethral tone and continence control. Therefore, the aim of the present study was to investigate the functional role of PDE (PDE-4, PDE-5 and PDE-1) isoenzymes in the isolated porcine urethral smooth muscle and mucosal layers. By including PDE-1 in this study, we aim to determine whether any effects observed for PDE-4 and PDE-5 inhibitors are truly isoenzyme-specific, rather than resulting from a broader, nonspecific PDE inhibition. Demonstrating distinct effects among these inhibitors would help confirm the specific roles of PDE-4 and PDE-5 in urethral smooth muscle. It is hypothesised that PDE-4 and PDE-5 inhibitors will differentially modulate urethral contractility through distinct interactions with the smooth muscle and mucosal layers.

## Materials and Methods

### Tissue Specimen Origin and Preparation

Female pig urinary bladders, with urethras attached were obtained from the local abattoir (Highchester Meats, Beaudesert) and immediately immersed in ice-cold Kreb’s bicarbonate solution (4 °C) composed of NaCl (118 mM), NaHCO_3_ (24.9 mM), glucose (11.7 mM), CaCl_2_ (2.5 mM), MgSO_4_ (1.2 mM), KCl (4.7 mM) and KH_2_PO_4_ (1.2 mM) and immediately transported to the laboratory for experimental procedures within 2 h. Each *n* in the experiments represents tissue obtained from a different animal to ensure variability.

From each animal, approximately 4–6 urethral tissue strips (7 mm × 4 mm) were prepared, with strips evenly distributed across the experimental groups (mucosa-intact smooth muscle, denuded smooth muscle and mucosal layer). Tissue strips were dissected from the proximal urethra segment, which was determined to be approximately 2 cm below the bladder neck. The mucosal (urothelium and lamina propria) layer was carefully removed from the smooth muscle layer using fine dissection under a microscope to ensure precision and minimal damage to the underlying smooth muscle. The contractile response to potassium chloride (80 mM) was assessed across all groups to verify the integrity of the smooth muscle function post-mucosa removal. Mucosa-intact tissue strips exhibited a contractile response of 5.92 ± 0.9 g (*n* = 56), which was comparable to mucosa-denuded strips (5.36 ± 1.2 g, *n* = 56). No significant differences were observed between the groups, confirming that mucosa removal did not impair the smooth muscle’s ability to contract in response to KCl (*p* > 0.05, Student’s paired *t*-test). Tissue strips were mounted longitudinally in 8 mL EZ-Bath organ baths (Global Towns Microtechnology, Sarasota, USA) containing Krebs-bicarbonate solution, maintained at 37 °C, and continuously gassed with 95% O_2_ and 5% CO_2_, with a pH of 7.4. A tension of 1.5–2 g was applied, and the tissue strips were allowed to equilibrate for 45 min with fresh solution washout every 15 min. The isometric tension developed by the tissues was recorded via a Powerlab recording system and Labchart software (ADInstruments, Castle Hill, NSW, Australia). Depending on the experiment, isometric tension was recorded for a total of approximately 1.5 h, including the washout period.

### Effects of PDE Inhibitors on Urethral Smooth Muscle Contractility

**Fig. 1:**
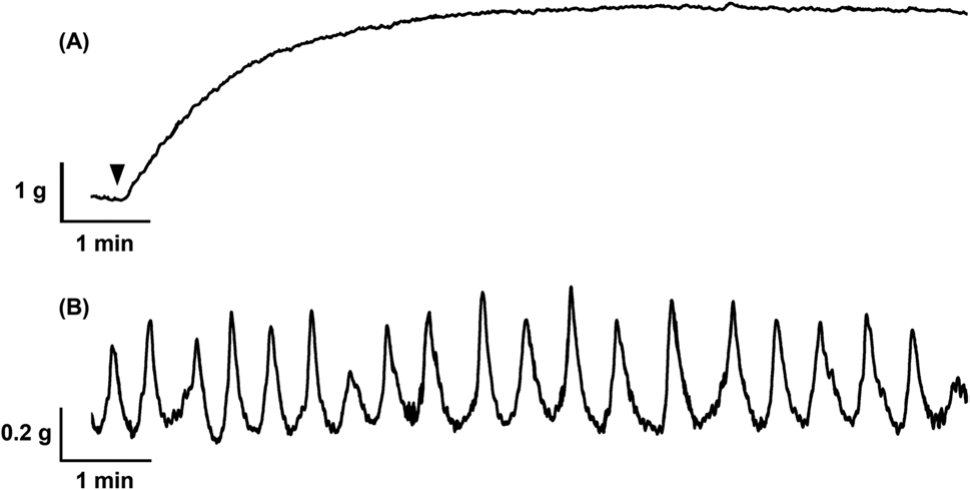
Representative traces of (**A**) urethral smooth muscle contractile response to a single concentration of phenylephrine (30 µM) and (**B**) urethral mucosal tissue developing spontaneous contractile activity

To investigate potential relaxatory effects of PDE-4 inhibitors rolipram and roflumilast, PDE-5 inhibitors sildenafil and tadalafil, and PDE-1 inhibitor vinpocetine on tonic contractile activity of urethral smooth muscle (mucosa-intact and denuded), tonic contractions were induced by α_1_-adrenoceptor agonist phenylephrine (30 μM) in tissue strips. During the storage phase of the micturition cycle, α_1_-adrenoceptor stimulation plays a role in maintaining urethral muscle tone by responding to sympathetic nervous system inputs. This helps prevent urinary leakage by ensuring the urethra can resist bladder pressure. Thus, phenylephrine is a suitable agent to induce tonic contractions in tissues strips as it mimics physiological tone mechanisms relevant to urinary incontinence. This precontractile agent provided a stable production of tonic activity in our experiments as has been reported in previous studies [15]. Upon generation of a consistent pattern of contractile activity (approximately 10 min) as shown in Fig. 1(B), one PDE inhibitor was cumulatively added at one time to all tissue strips in a tenfold serial dilution across the range of 0.1 nM to 10 µM. One tissue strip was only ever exposed to one PDE inhibitor. In a separate set of experiments, urethral smooth muscle tissue strips were incubated with nitric oxide (NO) donor sodium nitroprusside (SNP, 10 μm) for 30 min, followed by cumulative addition of tadalafil.

### Effects of PDE Inhibitors on Urethral Mucosal Strips Activity

In this part of the study, we aimed to investigate whether PDE inhibitors rolipram (PDE-4), roflumilast (PDE-4), sildenafil (PDE-5), tadalafil (PDE-5) and vinpocetine (PDE-1) affected the rate of spontaneous contractions generated by urethral mucosal tissue strips. Upon generation of a consistent pattern of spontaneous contractile activity, in the urethral mucosal tissue strips, one PDE inhibitor was cumulatively added at one time to all tissue strips (0.1 nM to 10 µM). One tissue strip was only ever exposed to one PDE inhibitor.

### Data and Statistical Analysis

In response to the agonist phenylephrine, isolated urethral smooth muscle tissue strips (denuded and mucosa-intact) developed tonic contractile activity (Fig. 1A). The relaxation of the tone of the contractions was measured over 3 min for each concentration of inhibitor (0.1 nM to 10 µM). Preliminary experiments suggested that the isolated urethral mucosal tissue strips do not respond tonically when exposed to phenylephrine (up to 100 µM, data not shown). During the equilibration period, these tissue strips generate a stable patten of spontaneous contractions (Fig. 1B). Thus, to measure the effects of PDE inhibitors on this activity, the rate of spontaneous contraction was measured across 3 min for each concentration of inhibitor (0.1 nM to 10 µM).

GraphPad Prism software (GraphPad, San Diego, USA) was used to perform all statistical analysis and graphical representation. Relaxant responses of urethral smooth muscle strips were expressed as the percentage of the decrease from plateaued contraction amplitude induced by phenylephrine (10 µM). All data were expressed as mean ± S.E.M. or S.D. of *n* preparations, where *n* is the number of animals. One-way ANOVAs followed by a Dunnett’s multiple comparisons test were performed to identify statistically significant differences, where *p* < 0.05 was considered statistically significant.

### Drugs and Chemicals

The chemicals used for the Krebs-bicarbonate solution were of analytical grade and purchased from Sigma-Aldrich (Castle Hill, Australia). Sodium nitroprusside and phenylephrine was obtained from Sigma-Aldrich (Castle Hill, Australia) and all PDE inhibitors (rolipram, roflumilast, sildenafil, tadalafil and vinpocetine) from Tocris (Noble Park, Australia). All drugs were dissolved in DMSO and subsequently, dH_2_0, except SNP which was dissolved in dH_2_O. All chemicals and drugs were prepared ensuring the highest concentration of DMSO never exceeded 0.5% in our final solutions, well within the range deemed noninfluential on smooth muscle physiology. Vehicle controls using DMSO at this concentration showed no significant effect on either phenylephrine-induced urethral smooth muscle contraction or the rate of spontaneous contraction.

## Results

All porcine urethral smooth muscle strips (*n* = 112) and mucosal tissue strips (*n* = 56) were allowed to equilibrate to a mean passive tension of 1.83 g ± 0.16 g and 1.53 ± 0.25 g, respectively. During the equilibration period, urethral smooth muscle strips remained quiescent. As depicted in Fig. 1(A), when these smooth muscle strips were subjected to phenylephrine (30 μM), they contracted tonically. The urethral mucosal strips developed a consistent and stable pattern of spontaneous activity during the equilibration period, as shown in Fig. 1(B), with a mean rate of 2.75 ± 0.89 (*n* = 56) contractions per minute.

### Effect of PDE Inhibitors on Urethral Smooth Muscle Contractility

**Fig. 2:**
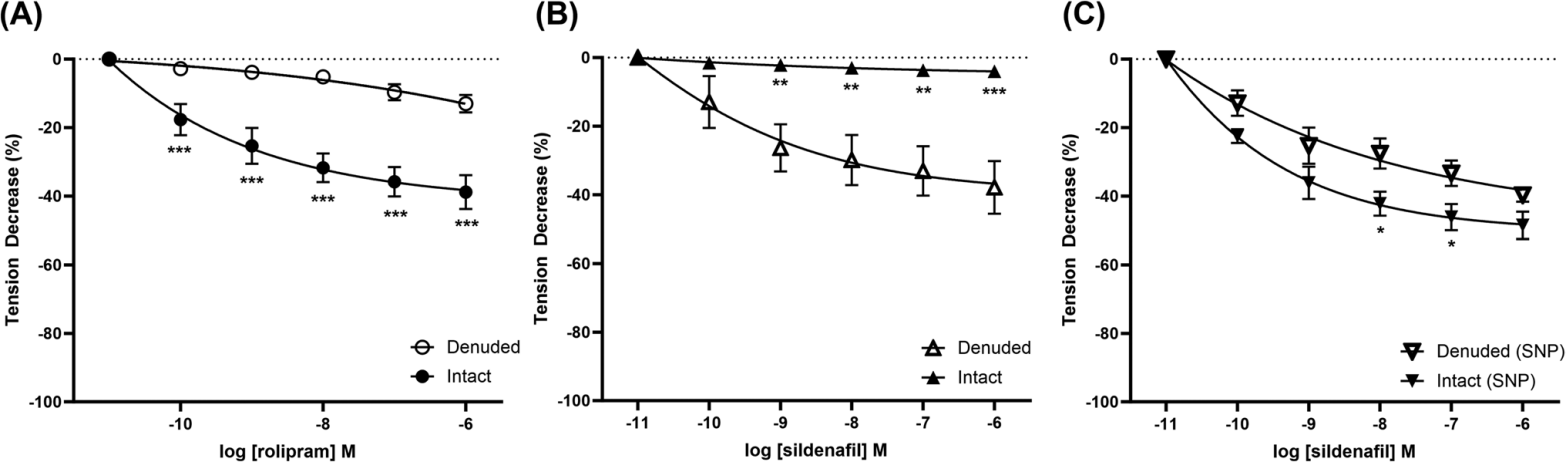
Concentration–response curves to PDE inhibitors (**A**) rolipram and sildenafil in the (**B**) absence and (**C**) presence of SNP in phenylephrine precontracted mucosa intact and denuded porcine urethral smooth muscle. Data represent the mean ± SEM (*n* = 8, *p < 0.05, **p < 0.01 and ***p < 0.001 vs denuded)

PDE-4 inhibitor rolipram concentration dependently decreased the phenylephrine-induced contractions in the isolated porcine urethral smooth muscle strips with intact mucosa (Fig. 2A). This relaxation was not observed in smooth muscle tissue strips denuded of the mucosa (Fig. 2A). Similar effects were observed when isolated tissue strips were exposed to increasing concentrations of PDE-4 inhibitor roflumilast (Table 1). PDE-5 inhibitor sildenafil concentration dependently attenuated phenylephrine-induced contractions in the isolated porcine urethral smooth muscle strips denuded of the mucosa, but had no effect on the strips with the mucosal layer intact (Fig. 2B). In the presence of nitric oxide donor sodium nitroprusside (SNP), the attenuation effect of sildenafil in the urethral smooth muscle strips denuded of mucosa was enhanced (Fig. 2C). Similar observations were found with PDE-5 inhibitor tadalafil (Table 1). PDE-1 inhibitor vinpocetine did not exert any significant effect on the urethral smooth muscle contractility (Table 1).

**Table 1:** Maximum relaxation of phenylephrine-induced contraction (%) in urethral smooth muscle strips (mucosa denuded and intact) by PDE inhibitors. Data represent mean ± SEM (*n* = 8)

	*Denuded*	*Intact*
*Rolipram*	5.74 ± 1.95	24.90 ± 5.87
*Roflumilast*	3.86 ± 1.27	20.81 ± 4.82
*Sildenafil*	23.35 ± 5.80	2.39 ± 0.61
*Sildenafil (SNP)*	23.11 ± 5.89	32.58 ± 7.54
*Tadalafil*	26.67 ± 7.06	3.36 ± 0.96
*Tadalafil (SNP)*	26.78 ± 6.91	31.43 ± 7.65
*Vinpocetine*	4.31 ± 1.54	4.28 ± 1.62

### Effects of PDE-4 and PDE-5 Inhibitors on Urethral Mucosa

**Fig. 3:**
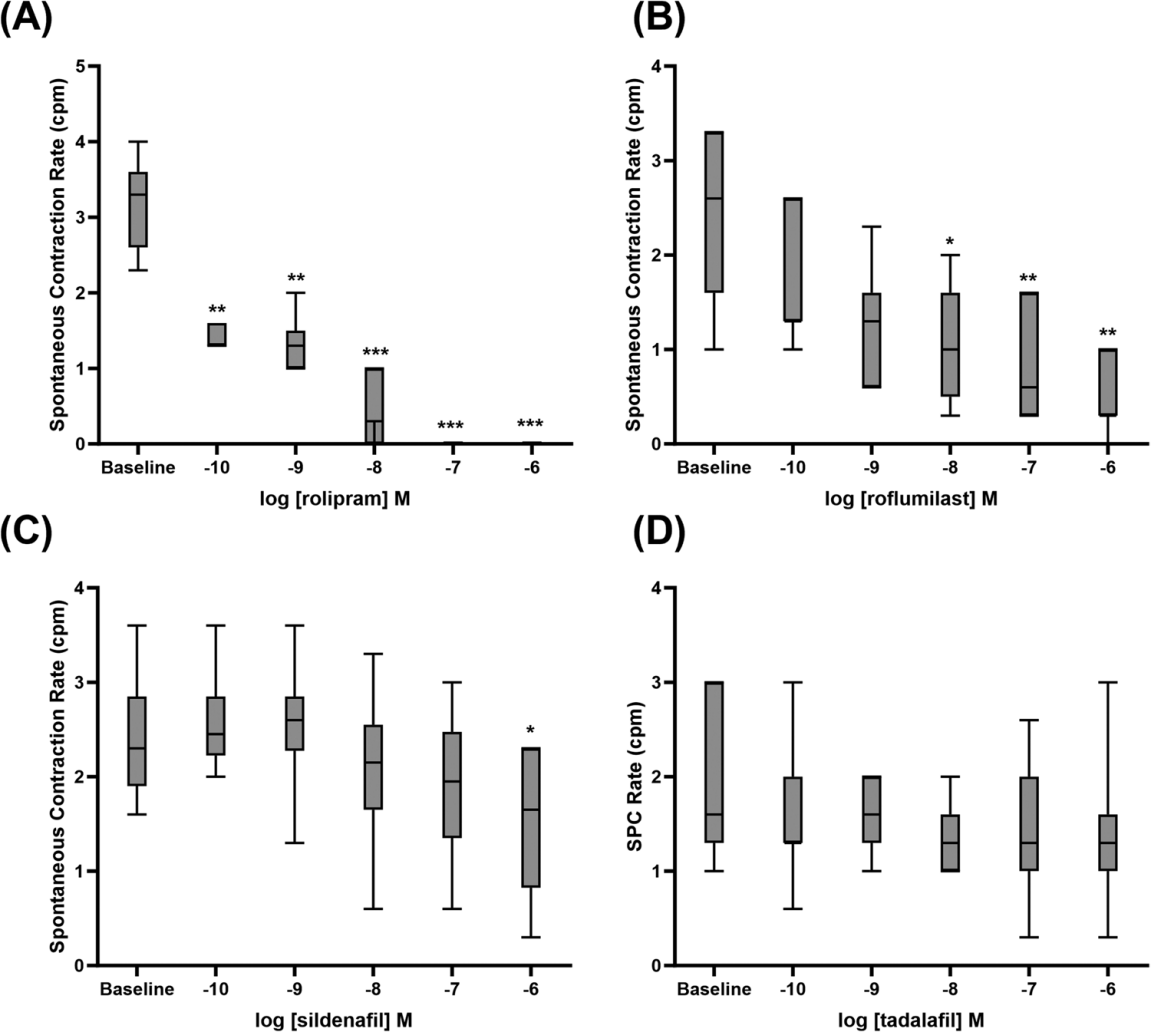
Spontaneous contraction rate (contractions per minute, cpm) of urethral mucosal strips in response to PDE inhibitors (**A**) rolipram, **(B)** roflumilast, (**C**) sildenafil and (**D**) tadalafil. Data represent the mean ± SD (*n* = 8, *p < 0.05, **p < 0.01 and ***p < 0.001 vs baseline)

In the urethral mucosal strips, rolipram and roflumilast were capable of significantly reducing the rate of spontaneous contraction significantly, starting from concentrations of 0.1 nM and 10 nM, respectively, while sildenafil had a significant depressant effect at the highest concentration used, 1 µM (Fig. 3A, B, and C). Tadalafil (Fig. 3D) and vinpocetine (data not shown) did not affect the spontaneous contraction rate of these tissues.

## Discussion

The present study aimed to investigate the functional role of PDE isoenzymes in the isolated porcine urethral smooth muscle and mucosal layers. This is the first functional study on the urethra that differentiates the role of the enzyme within different layers of the tissues (smooth muscle and mucosal layer). Our findings demonstrate that (1) PDE-4 inhibition effectively relaxed mucosa-intact urethral smooth muscle tissue strips, (2) reduced the rate of spontaneous contractions in the urethral mucosal strips, while (3) PDE-5 inhibition relaxed urethral smooth muscle tissue strips denuded of the mucosa, and (4) did not alter the rate of spontaneous contraction in the urethral mucosal tissue strips.

The present findings demonstrate that both PDE-4 inhibitors rolipram and roflumilast attenuated phenylephrine-induced tonic contractions in the isolated urethral smooth muscle strip with mucosa intact. These results are in line with a previous study that reported the role of PDE-4 inhibitors in the relaxation of the human urethral smooth muscle [15]. The effectiveness of rolipram and roflumilast can be attributed to the ability of these inhibitors to increase in intracellular cAMP levels leading to activation of subsequent messenger molecules resulting in the relaxation of the urethral smooth muscle [21]. Interestingly, when the mucosal layer was removed, the attenuation effect of the PDE-4 inhibition was not present, suggesting that the mucosa may possess attributes resulting in the significant depressant effect observed. This is potentially related to the significant reduction of spontaneous contraction rate in the urethral mucosal strips induced by both rolipram (0.1 nM and above) and roflumilast (10 nM and above). This is the first study to demonstrate that the mucosal layer of the urethra also exhibits spontaneous contraction activity like the bladder. While the exact mechanisms as to its purpose are yet to be elucidated, a previous study suggested that these spontaneous contractions are likely generated by urethral interstitial cells found in the lamina propria layer of the mucosa [22]. Overall, these results suggest that the PDE-4 isoenzyme is involved in the modulation of mucosal functions, and this affects its role in modulating urethral smooth muscle contraction.

PDE-5 inhibition did not alter the spontaneous contraction rate of the urethral mucosa except at the highest concentration (1 µM), where it is unlikely to be selective for PDE-5. Therefore, it is proposed that in the urethra, the cAMP pathway predominantly modulates mucosal functions rather than the cGMP pathway. Additionally, our findings suggest that PDE-5 inhibition dose-dependently relaxed urethral smooth muscle tissue strips at similar levels in the absence and presence of the NO donor SNP. These findings support observations reported from a previous study where sildenafil relaxed the isolated human urethral smooth muscle [18], and that the NO/cGMP pathway can modulate the functions of these tissues [15]. Interestingly, for mucosa-intact smooth muscle strips, sildenafil was not as effective in relaxing the phenylephrine-induced contractions in the absence of the NO donor. Similar findings were found in the rat tissue [17], although the study did not indicate whether the mucosa was intact or denuded. The present findings suggest that the mucosa may prevent endogenous NO production in the tissue. There is a possibility of crosstalk between cGMP and cAMP pathways, as have been shown to occur in the myocardiocytes of the heart [23]. However, unlike in the heart where cGMP can influence the intracellular levels of cAMP through regulation of the activity of cAMP-hydrolysing PDEs [23, 24], our findings propose that in the urethra, the cAMP pathway predominantly modulates the mucosal functions, which may inhibit NO production affecting the cGMP pathway. The present findings suggest that the presence of the mucosa (which is the physiologically relevant condition) prevents relaxation induced by PDE-5 inhibitors. These inhibitors have shown effectiveness in reducing lower urinary tract symptoms in multiple clinical trials, likely due to relaxation of the detrusor muscle. Thus, we propose that the mucosal layer protects the urethral smooth muscle from relaxing, which would counteract the desired effects of PDE-5 inhibitors in these circumstances.

The current study had some limitations. First, the use of a porcine urethra as a model does not fully represent the underlying physiology of human urethral tissue in relation to the contractile properties of human urethral tissue. Further studies using human urethral tissue would be required to establish clinical relevancy. Second, investigations into different PDE isoenzymes will also be beneficial to identify more specific targets. Lastly, the mechanisms underlying the effects of PDE inhibitors on the control of the urethra remain unclear, especially within different layers of the urethra. The potential of a cross-talk between the cAMP and cGMP pathway within the urethra can continued to be investigated through measurements of cAMP and cGMP levels within the different layers and under different circumstances.

## Conclusions

The present study’s findings add to the limited body of knowledge on the role of PDE isoenzymes in urethral smooth muscle and mucosal functions. In summary, our findings demonstrate that PDE-4 inhibitors can effectively relax mucosa-intact urethral smooth muscle tissue strips and reduce the rate of spontaneous contraction in the urethral mucosal strips. PDE-5 inhibitors can effectively relax urethral smooth tissue strips but rely on an exogenous NO source in the presence of the mucosa.

## Data Availability

Data can be made available upon reasonable request.

## Abbreviations

cAMP: Cyclic adenosine monophosphate
cGMP: Cyclic guanosine monophosphate
NO: Nitric oxide
PDE: Phosphodiesterase
SNP: Sodium nitroprusside
SUI: Stress urinary incontinence
